# Mental health care during the Ebola virus disease outbreak in Sierra Leone

**DOI:** 10.2471/BLT.16.190470

**Published:** 2017-10-31

**Authors:** Stania Kamara, Anna Walder, Jennifer Duncan, Antoinet Kabbedijk, Peter Hughes, Andrew Muana

**Affiliations:** a King’s Sierra Leone Partnership, 2.04 Weston Education Centre, 10 Cutcombe Road, London SE5 9RJ, England.; b Connaught Hospital, Ministry of Health and Sanitation, Government of Sierra Leone, Freetown Sierra Leone.; c Sierra Leone Psychiatric Hospital, Ministry of Health and Sanitation, Government of Sierra Leone, Freetown, Sierra Leone.

## Abstract

**Problem:**

Reported levels of mental health and psychosocial problems rose during the 2014–2015 Ebola virus disease outbreak in Sierra Leone.

**Approach:**

As part of the emergency response, existing plans to create mental health units within the existing hospital framework were brought forward. A nurse-led mental health and psychosocial support service, with an inpatient liaison service and an outpatient clinic, was set up at the largest government hospital in the country. One mental health nurse trained general nurses in psychological first aid, case identification and referral pathways. Health-care staff attended mental well-being workshops on coping with stigma and stress.

**Local setting:**

Mental health service provision in Sierra Leone is poor, with one specialist psychiatric hospital to serve the population of 7 million.

**Relevant changes:**

From March 2015 to February 2016, 143 patients were seen at the clinic; 20 had survived or had relatives affected by Ebola virus disease. Half the patients (71) had mild distress or depression, anxiety disorders and grief or social problems, while 30 patients presented with psychosis requiring medication. Fourteen non-specialist nurses received mental health awareness training. Over 100 physicians, nurses and auxiliary staff participated in well-being workshops.

**Lessons learnt:**

A nurse-led approach within a non-specialist setting was a successful model for delivering mental health and psychosocial support services during the Ebola outbreak in Sierra Leone. Strong leadership and partnerships were essential for establishing a successful service. Lack of affordable psychotropic medications, limited human resources and weak social welfare structures remain challenges.

## Résumé

**Problème:**

Les taux de problèmes mentaux et psychosociaux signalés ont augmenté pendant la flambée de maladie à virus Ebola qui a sévi en 2014-2015 en Sierra Leone.

**Approche:**

Durant l'intervention d'urgence, des projets visant à créer des unités de santé mentale dans le cadre hospitalier existant ont été examinés. Un service de soutien psychosocial et de santé mentale, dirigé par du personnel infirmier et doté d'un service de liaison avec le milieu hospitalier et d'un service de consultation externe, a été mis en place dans le plus grand hôpital public du pays. Une infirmière spécialisée en santé mentale a formé des infirmières générales aux premiers secours psychologiques, à l'identification des cas et aux parcours de prise en charge. Le personnel de santé a assisté à des ateliers sur le bien-être mental destinés à apprendre à faire face à la stigmatisation et au stress.

**Environnement local:**

L'offre de services de santé mentale est faible en Sierra Leone, avec un hôpital psychiatrique spécialisé pour 7 millions de personnes.

**Changements significatifs:**

De mars 2015 à février 2016, 143 patients ont été reçus en consultation; 20 avaient survécu ou avaient des proches touchés par la maladie à virus Ebola. La moitié des patients (71) souffraient de détresse légère ou de dépression, de troubles anxieux et de chagrin ou de problèmes sociaux, tandis que 30 patients présentaient une psychose qui a nécessité un traitement médicamenteux. Quatorze infirmières non spécialisées ont suivi une formation de sensibilisation à la santé mentale. Plus de 100 médecins, infirmières et membres du personnel auxiliaire ont participé aux ateliers sur le bien-être mental.

**Leçons tirées:**

Cette démarche, menée par du personnel infirmier dans un environnement non spécialisé, a permis de fournir des services de soutien psychosocial et de santé mentale pendant la flambée de maladie à virus Ebola en Sierra Leone. Une direction forte et des partenariats solides ont été essentiels à la mise en place de ces services. Le prix élevé des psychotropes, les ressources humaines limitées et le manque de structures de protection sociale demeurent problématiques.

## Resumen

**Situación:**

Se informó de un aumento de los problemas de salud mental y de tipo psicosocial durante el brote de la enfermedad del virus del Ébola en los años 2014 y 2015 en Sierra Leona.

**Enfoque:**

Como parte de la respuesta de emergencia, se presentaron los planes para crear unidades de salud mental dentro del marco hospitalario existente. Se estableció un servicio de salud mental y de apoyo psicosocial dirigido por enfermeras, con un servicio de enlace hospitalario y una clínica ambulatoria, en el hospital gubernamental más grande del país. Una enfermera de salud mental formó a enfermeras sin especialización en primeros auxilios psicológicos, identificación de casos y vías de derivación. El personal de salud asistió a talleres de bienestar mental sobre cómo lidiar con el estigma y el estrés.

**Marco regional:**

La provisión de servicios de salud mental en Sierra Leona es deficiente, con un hospital psiquiátrico especializado para atender a la población de 7 millones.

**Cambios importantes:**

Entre marzo de 2015 y febrero de 2016, la clínica atendió a 143 pacientes, de los cuales 20 habían sobrevivido o tenían familiares afectados por la enfermedad del virus del Ébola. La mitad de los pacientes (71) sufrieron trastornos o depresiones leves, trastornos de ansiedad y duelo o problemas sociales, mientras que 30 pacientes presentaron una psicosis que requirió medicación. Catorce enfermeras no especializadas recibieron formación en sensibilización sobre salud mental. Más de 100 médicos, enfermeras y personal auxiliar participaron en talleres de bienestar.

**Lecciones aprendidas:**

Un enfoque dirigido por enfermeras dentro de un entorno no especializado fue un modelo de éxito para ofrecer servicios de salud mental y apoyo psicosocial durante el brote de Ébola en Sierra Leona. El fuerte liderazgo y las sólidas colaboraciones fueron esenciales para constituir un servicio de éxito. La falta de medicamentos psicotrópicos asequibles, los recursos humanos limitados y las débiles estructuras de bienestar social siguen presentando un desafío.

## ملخص

**المشكلة:**

ارتفعت مستويات مشكلات الصحة العقلية والمشكلات النفسية الاجتماعية التي جرى تسجيلها خلال الفترة 2014 – 2015 في وقت تفشي فيروس الإيبولا في سيراليون.

**الأسلوب:**

تم في سياق الاستجابة لحالات الطوارئ طرح خطط متوفرة لإنشاء وحدات للصحة العقلية ضمن الإطار الحالي للمستشفيات. وتم تنظيم خدمات الصحة العقلية والدعم النفسي الاجتماعي المقدم من جانب الممرضين بالإضافة إلى التنسيق مع المرضى المقيمين بالمستشفيات والمترددين على العيادة الخارجية، وذلك في أكبر المستشفيات الحكومية في البلاد. وقام أحد الممرضين العاملين في مجال الصحة العقلية بتدريب ممرضي الخدمة الطبية العامة لتقديم خدمات الإغاثة الأولية فيما يتعلق بالمشكلات النفسية الاجتماعية والتعرف على الحالات المرضية والخطوات المقررة لإحالة المرضى للمختصين. وحضر العاملون في مجال الرعاية الصحية حلقة عمل في مجال الصحة والراحة النفسية تتناول التكيف مع الضغوط النفسية والمعاناة من علامات المرض الظاهرة.

**المواقع المحلية:**

وتعاني سيراليون من نقص في الموارد اللازمة لتوفير خدمات الصحة العقلية، حيث يوجد لديها مستشفى نفسي متخصص واحد يخدم مجموعة من السكان يبلغ عددها 7 ملايين نسمة.

**التغيّرات ذات الصلة:**

شهدت الفترة من مارس/آذار 2015 حتى فبراير/شباط 2016 تواجد 143 مريضًا في العيادة الطبية؛ وظل 20 مريضًا على قيد الحياة أو كان لديهم أقرباء تأثروا بمرض فيروس الإيبولا. وعانى نصف المرضى (71) من اكتئاب أو ضائقة نفسية خفيفة الأثر، أو اضطرابات القلق والجزع، أو واجهوا مشكلات اجتماعية، بينما ظهرت أعراض الذهان التي تتطلب العلاج لدى 30 مريضًا. وتلقى أربعة عشر ممرضًا غير متخصص تدريبًا توعويًا في مجال الصحة العقلية. وشارك ما يزيد عن 100 فرد من الأطباء والممرضين والمساعدين لهم في حلقات العمل في مجال الصحة والراحة النفسية.

**الدروس المستفادة:**

كان النهج المتبع في محيط غير المتخصصين تحت إشراف الممرضين نموذجًا ناجحًا لتقديم خدمات الصحة العقلية والدعم النفسي الاجتماعي أثناء ظهور مرض الإيبولا في سيراليون. ولعبت القيادة والشراكات القوية دورًا جوهريًا في تنظيم خدمة ناجحة. وتظل التحديات قائمة، إذ تتمثل في النقص في العلاج المؤثر على العقل والموارد البشرية المحدودة وضعف مستوى الهيئات الاجتماعية التي تعمل على تحقيق الرخاء.

## 摘要

**问题:**

塞拉利昂 2014 年至 2015 年埃博拉病毒疫情暴发期间报告的精神健康和心理问题等级上升。

**方法:**

作为应急响应的一部分，当时提出了在现有医院组织框架内创建精神健康小组的现行计划。在该国规模最大的政府医院内设立由护士主导的精神健康和心理支持服务，提供住院患者联络和门诊服务。由一位精神健康护士在心理急救、病例鉴定和转诊途径方面对普通护士进行培训。医疗护理人员参加了关于如何应对羞辱和压力的精神健康研讨会。

**当地状况:**

塞拉利昂精神健康服务匮乏，由一家精神病专科医院为 700 万人提供服务。

**相关变化:**

从 2015 年 3 月到 2016 年 2 月，143 位患者前往门诊就医；其中 20 位本人感染过埃博拉病毒或亲属受到过埃博拉病毒疫情的影响。一半的患者 (71) 存在轻微的痛苦、抑郁、焦虑、忧伤或社交问题，30 位患者出现需要药物治疗的精神病。十四位非专科护士接受了精神健康意识培训。100 多位医生、护士和辅助人员参加了健康研讨会。

**经验教训:**

在塞拉利昂埃博拉暴发期间，非专业环境中由护士主导的方法是一种成功提供精神健康和心理支持服务的模式。强有力的领导与合作伙伴关系对于创建成功的服务至关重要。缺乏经济实惠的精神治疗药物、有限的人力资源以及社会福利薄弱环节依然是挑战。

## Резюме

**Проблема:**

Зарегистрированные уровни проблем психического здоровья и психосоциальных проблем повысились в период вспышки инфекции, вызываемой вирусом Эбола, в Сьерра-Леоне в 2014–2015 годах.

**Подход:**

В рамках экстренного реагирования были предложены уже существующие планы по созданию подразделений по охране психического здоровья в рамках существующей больничной базы. В крупнейшей государственной больнице в стране была создана сестринская служба по охране психического здоровья и психосоциальной поддержке, имеющая стационарную службу связи и амбулаторную клинику. Одна медсестра службы по охране психического здоровья обучила медсестер общего профиля предоставлению психологической помощи, идентификации случаев и направлению к специалистам. Сотрудники здравоохранения посетили семинары по охране психического здоровья, посвященные борьбе со стигмой и стрессом.

**Местные условия:**

Уровень медицинского обслуживания при психических заболеваниях в Сьерра-Леоне низкий, при этом одна специализированная психиатрическая больница обслуживает население в 7 миллионов человек.

**Осуществленные перемены:**

С марта 2015 года по февраль 2016 года в клинике наблюдались 143 пациента, 20 из них выжили или имели родственников, инфицированных вирусом Эбола. У половины пациентов (71) были состояния, характеризующиеся как умеренный стресс или депрессия, тревожные расстройства и горе или социальные проблемы, в то время как у 30 пациентов был психоз, требующий лечения. Четырнадцать неспециализированных медсестер прошли обучение по вопросам психического здоровья. В семинарах по психическому здоровью приняли участие более 100 врачей, медсестер и вспомогательного персонала.

**Выводы:**

Подход, основанный на привлечении медсестер в условиях отсутствия узких специалистов, являлся успешной моделью для оказания медицинской помощи при психических расстройствах и психосоциальной поддержки во время вспышки инфекции, вызываемой вирусом Эбола, в Сьерра-Леоне. Сильное руководство и партнерские отношения имеют важное значение для создания успешной службы по охране психического здоровья. Отсутствие доступных психотропных препаратов, условия ограниченных человеческих ресурсов и слабая система социального обеспечения все еще остаются проблемой.

## Introduction

The impact of complex humanitarian emergencies on the mental health and psychosocial well-being of the population is multi-layered and endures long after the emergency.^1^ Studies have demonstrated that mental health and psychosocial support responses in emergency settings are often poorly coordinated, not evidence-based and not implemented within formal national frameworks.^2^ Research highlights the importance of cultural understanding, training, assessment, monitoring and evaluation.^3^^,^^4^ *The Sphere handbook*: *humanitarian charter and minimum standards in humanitarian response* ^5^ and the *Inter-Agency Standing Committee (IASC) Guidelines for mental health and psychosocial support in emergency settings *provide standards on such implementation.^1^

In May 2014, the first Ebola virus disease case was declared in Sierra Leone; a total of 8700 people were infected and 3600 died. Sierra Leone was declared Ebola-free in November 2015 with 5100 recorded survivors^6^ and 3400 orphaned children.^7^ During the outbreak, anecdotal evidence was that increased numbers of people reported mental health and psychosocial problems.^8^ The outbreak affected existing health structures, halted routine activities and had a major impact on the health workforce. Mortality among health-care workers was 69% (152/219) and they were 20–30 times more likely than the general population to contract Ebola.^9^ Hospital staff especially faced stigmatization, blame and social exclusion and there were high levels of absenteeism from work.

## Local setting

Mental health service provision in Sierra Leone is poor. In 2009 an estimated 2058 people received some form of mental health treatment, out of about 102 000 people (3% of the 3.4 million adult population) who had a severe mental disorder.^10^ There is one specialist psychiatric hospital in the country, located in the capital Freetown, to serve the population of 7 million.

During the Ebola virus outbreak, the Sierra Leone psychiatric hospital was closed to admissions to prevent disease transmission. Existing government plans to create new decentralized mental health units across the country^11^ were brought forward as part of the emergency response. Mental health nurses who had received 12–18 months’ mental health training in 2012–2013 from a bespoke nursing curriculum^12^ were deployed to general hospitals in various districts. We describe here our experience of establishing one of the new units ‒ a nurse-led mental health and psychosocial support service at Connaught hospital in Freetown, the largest government hospital in the country with approximately 300 beds.

## Approach

King’s Sierra Leone Partnership, which was already supporting the government’s mental health strategic plan, assisted with the development of the unit at Connaught hospital. To equip the nurses, the World Health Organization (WHO), CBM International and local partners provided the nurses with psychological first-aid training^13^ focused on supporting those affected by Ebola virus disease.

Meetings were held at Connaught hospital with the mental health focal person from the health ministry, the hospital management team, the mental health nurse allocated to the hospital and the King’s Sierra Leone Partnership team. The agreed objective was to create an inpatient liaison service and an outpatient clinic for community access. This would be a sustainable service, integrated into the existing hospital framework and providing mental health and psychosocial support for all, including those affected by Ebola. The health ministry met the human resources costs. The hospital provided office and clinical space and funding for consumables. King’s Sierra Leone Partnership provided technical expertise, staff supervision and office equipment.

The service was launched in March 2015 and was available to those living within the Freetown city area (about 1 million people) or anyone admitted to Connaught hospital. The partnership devised a standard operating procedure. Individuals of any age with a known or suspected mental health problem or psychosocial need met the referral criteria. A service level agreement with the Sierra Leone psychiatric hospital allowed transfers for inpatient care. In keeping with hospital protocol a registration fee was levied and waived if service users were unable to pay. A single mental health nurse provided the service, with prescribing of medication carried out by a linked hospital medical physician. A range of treatments were provided. Psychological interventions were the most common, comprising basic counselling and problem-solving therapy. The WHO Mental Health Gap Action Programme (mhGAP) intervention guide,^14^ was the model of care used. A proforma for initial assessment of patients (including demographic information, psychiatric and risk assessment) was created. Monthly monitoring and evaluation data were collected manually from the clinic ledger and presented to the hospital and health ministry management teams.

To strengthen the skills of Connaught hospital’s non-specialist nurses, mental health awareness training was provided by the mental health nurse and King’s Sierra Leone Partnership volunteer. A half-day session on psychological first aid,^13^ case identification and referral pathways was delivered to a group of 14 ward nurses.

Mental wellbeing workshops were held for nurses, auxiliary staff and physicians who worked at Connaught hospital, including those working within the Ebola holding unit. These workshops were created and led by the mental health nurse and comprised a series of half-day sessions, for groups of 10‒15, on coping with stigma and discrimination, stress management and self-care. The mental health nurse provided one-to-one counselling to staff requiring more support.

The human immunodeficiency virus (HIV) and epilepsy services at Connaught hospital were also offered half-day mental health awareness training by the mental health nurse, and referral pathways were created across the services. Partnerships were established with service user groups (e.g. the HIV peer network), national and international nongovernmental organizations (NGOs) providing livelihood support, child protection organizations and faith groups.

A King’s Sierra Leone Partnership volunteer (senior mental health nurse or psychiatrist) provided regular supervision and mentoring. Weekly individual supervision of the local mental health nurse focused on clinical case review, service monitoring and continuous professional development. Monthly peer supervision including other mental health nurses in Freetown focused on clinical case review, sharing of resources (e.g. information about livelihood support programmes) and continuous professional development. The mhGAP guide was used in supervision to support case-based discussion learning and to reinforce its application within clinical practice.

Challenges facing the service were addressed during weekly mental health team meetings (attended by the mental health nurse and King’s Sierra Leone Partnership volunteer). A timetable including times for home visits, clinics, inpatient work and supervision helped the mental health nurse to manage the workload.

## Relevant changes

A total of 143 patients were seen within the first 12 months of the service from March 2015 to February 2016 (Table 1). Most patients (96; 67%) were referred from another department at Connaught hospital and 7 (5%) were referred from Ebola clinics; 17 (12%) were referred by themselves, or by family or other relatives.

**Table 1:** Characteristics and outcomes of patients attending the Connaught hospital psychosocial and counselling clinic, Sierra Leone, March 2015–February 2016

Characteristics	No. (%) of patients (*n* = 143)
**Sex**	
Male	68 (48)
Female	75 (52)
**Age, years**	
0–17	27 (19)
18–34	64 (45)
35–54	33 (23)
55–74	15 (10)
75+	2 (< 1)
Unknown	2 (< 1)
**Referral source**	
Self, family or relatives	17 (12)
Connaught hospital department	96 (67)
Ebola disease holding unit or treatment centre	6 (4)
Ebola disease survivor clinic^a^	1 (< 1)
Nongovernmental organization^b^	15 (10)
Other	8 (6)
**Ebola virus disease status**	
Survived infection	7 (5)
Relative died or survived infection	13 (9)
Not directly affected	123 (86)
**Diagnosis^c^**	
Epilepsy or seizures	10 (7)
Alcohol or other substance use disorder	1 (< 1)
Intellectual disability	7 (5)
Psychotic disorder (including mania)	30 (21)
Moderate to severe emotional disorder or depression	17 (12)
Other psychological complaint	71 (50)
Medically unexplained somatic complaint	5 (3)
No mental disorder	2 (1)^d^
**Intervention^e^**	
Psychotropic medication	34 (15)
Psychological intervention	141 (61)
Social intervention	58 (25)
**Outcome**	
Referred to Sierra Leone psychiatric hospital (for inpatient mental health care)	1 (< 1)
Discharged from care	95 (66)
Remained on caseload of clinic	47 (33)

^a^ Clinics established by Ebola holding units and treatment centres for follow-up of survivors after discharge.

^b^ Including Médecins Sans Frontières, GOAL and human immunodeficiency virus peer networks.

^c^ According to case definitions of the United Nations High Commissioner for Refugees’ health information system.^15^

^d^ One patient was classified as malingering, the other had housing issues only.

^e^ Patients could have more than one intervention.

The most common diagnostic category was mild distress or depression, anxiety disorders and grief or social problems. Thirty patients (21%) presented with psychosis requiring medication. During the Ebola outbreak, an international NGO provided some medicines (e.g. haloperidol and amitriptyline) which were allocated to those unable to pay. Some service users reported accessing alternative treatment (including traditional and faith healing) when medication was not available.

Seven of the patients (5%) had survived Ebola virus disease and 13 (9%) were relatives of the deceased or survivors. Survivors and bereaved relatives presented with normal grief or mild depressive or anxiety symptoms and often reported being stigmatized or discriminated against within their communities. Those who lost family income earners experienced financial difficulties.

Fourteen non-specialist nurses were trained in mental health awareness and provided basic support on their wards and referred patients to the service. Over 100 Connaught hospital nurses, auxiliary staff and physicians participated in mental wellbeing workshops.

Monthly updates to the hospital management encouraged service improvements. From March 2015 to February 2016, approximately 30 abandoned patients (those with no relatives to provide care or financial support) were referred to the service. Evidence of high use by abandoned patients led to a successful request for a social worker to be deployed to the hospital.

## Lesson learnt

Early engagement of participants and a partnership approach with clear roles and responsibilities for all parties was key to ensuring ownership of and commitment to the service (Box 1). The health ministry and the hospital management responded positively to mental health and psychosocial support services being incorporated into a general hospital. Shared supervision was essential for maintaining clinical standards, developing competencies and providing a support network for the mental health nurses. The mental health service at the hospital is effective, integrated and has strengthened local capacity. People are now able to access affordable mental health care at a general hospital.

Box 1 **Summary of main lessons learnt**

- A nurse-led approach within a non-specialist setting was a successful model for delivering mental health and psychosocial support services during the Ebola virus disease outbreak in Sierra Leone.
- Strong leadership and partnerships between the health ministry and mental health nurses, nongovernmental organizations and hospital management were essential for establishing a successful service.
- Lack of affordable psychotropic medications, limited human resources and weak social welfare structures remain key challenges to care delivery.

The service’s ability to adapt and respond to changing needs ensured that support for health-care workers could be provided as the impact of the Ebola disease workload became apparent. The service provided care not only for survivors, but all those affected by the outbreak who presented with psychosocial needs.

There were challenges too. Although limited supplies of antipsychotic medications were available in local pharmacies, some patients could not afford them. The workload was high for a single nurse and the mental health nurse faced a risk of burnout and fatigue. Most referrals were from within Connaught hospital. We suspect community uptake was low because the service was new and the community had previous experience of mental health services at the hospital. Staff recruitment and training and community uptake therefore remain areas for development. Much of the focus has been on providing care for Ebola survivors, drawing attention and resources away from mental health services for the wider population.

The Ebola virus disease outbreak weakened an already fragile health system and disrupted existing plans to develop mental health services across the country. However, the emergency response provided the opportunity, resources and focus necessary to create the new units.^16^ Our experience has guided the establishment of 14 other mental health units countrywide so far. The service is inclusive and accessible to the entire population. There are plans to further develop the service, with integration into primary-care structures, increased community utilization and greater staff recruitment. A service evaluation ‒ measuring outcomes, follow-up rates, barriers to access and service coverage ‒ is underway. We believe our approach is a suitable framework for delivering mental health services and developing more resilient systems during an emergency response.
